# Assessing local vulnerability to climate change in Ecuador

**DOI:** 10.1186/s40064-015-1536-z

**Published:** 2015-11-26

**Authors:** Mario Andres Fernandez, Santiago J. Bucaram, Willington Renteria

**Affiliations:** Governance and Policy Team, Landcare Research, Auckland, New Zealand; School of Management and Economics, Universidad San Francisco de Quito, Quito, Ecuador; Instituto Oceanográfico de la Armada, Guayaquil, Ecuador

**Keywords:** Exposure, Sensitivity, Adaptive capacity, Indicator, Empirical orthogonal function

## Abstract

Vulnerability assessments have become necessary to increase the understanding of climate-sensitive systems and inform resource allocation in developing countries. Challenges arise when poor economic and social development combines with heterogeneous climatic conditions. Thus, finding and harmonizing good-quality data at local scale may be a significant hurdle for vulnerability research. In this paper we assess vulnerability to climate change at a local level in Ecuador. We take Ecuador as a case study as socioeconomic data are readily available. To incorporate the spatial and temporal pattern of the climatic variables we use reanalysis datasets and empirical orthogonal functions. Our assessment strategy relies on the statistical behavior of climatic and socioeconomic indicators for the weighting and aggregation mechanism into a composite vulnerability indicator. Rather than assuming equal contribution to the formation of the composite indicator, we assume that the weights of the indicators vary inversely as the variance over the cantons (administrative division of Ecuador). This approach captures the multi-dimensionality of vulnerability in a comprehensive form. We find that the least vulnerable cantons concentrate around Ecuador’s largest cities (e.g. Quito and Guayaquil); however, approximately 20 % of the national population lives in other cantons that are categorized as highly and very highly vulnerable to climate change. Results also show that the main determinants of high vulnerability are the lack of land tenure in agricultural areas and the nonexistence of government-funded programs directed to environmental and climate change management.

**Electronic supplementary material:**

The online version of this article (doi:10.1186/s40064-015-1536-z) contains supplementary material, which is available to authorized users.

## Background

The Intergovernmental Panel on Climate Change (IPCC) defines vulnerability as the degree to which geophysical, biological, and socio-economic systems are susceptible to, and unable to cope with adverse impacts of climate change (Houghton 1996). As these impacts at local scales are uncertain, vulnerability assessments have become necessary (Adger et al. 2004) to increase the understanding of climate-sensitive systems and to inform the specification of targets and allocation of funds. Other uses for assessments are to prioritize political and research efforts, to develop and to implement adaptation strategies (Füssel and Klein 2006); and to evaluate program/policy effectiveness in data-scarce regions (Adger et al. 2004; Pandey and Jha 2011).

The evolution of vulnerability assessments has revealed trends toward interdisciplinary analyses of the consequences of climate change, as well as integration with adaptation, environmental degradation, agricultural productivity, food security, population growth, and conflict research (Füssel and Klein 2006; Schilling et al. 2012).

Though there is no uniform methodology to assess vulnerability and its components (i.e. exposure, sensitivity and adaptive capacity), all vulnerability assessments require a detailed contextual understanding of the relevant systems and of the multiple stressors (Füssel 2010). Assessments have been conducted at different geographic scales, such as watersheds, rural areas (Eakin 2005), regions, countries, or worldwide (Pachauri et al. 2014). These assessments have made use of a wide array of technical tools such as local-level case studies (O’Brien et al. 2004; Sutanta et al. 2013), resilience indicators (Brenkert and Malone 2005), bio-economic models (Schilling et al. 2012), general equilibrium models (Parry et al. 2004; Tol 2002), and cross-sectional studies (Mendelsohn et al. 2006). The selection of the appropriate approach depends on the research or policy questions addressed, the urgency of the threat, the geographical and temporal scope of the analysis, the reliability of future climate impact projections, the level of previous knowledge, and the availability of data, expertise, and other resources (Füssel and Klein 2002, 2006; Füssel 2010).

Regardless of the assessment approach, challenges arise when the regions analyzed are communities that suffer from food, health and environmental insecurity, poverty, economic inequalities, weak governance, deficient infrastructure and education, lack of access to appropriate resources, or poor capacity to deal with extreme events (Bele et al. 2013). These issues are likely to determine the sensitivity of communities to climate change (Watson et al. 1998). For example, poverty and inequality are associated with poor quality housing, which is easily damaged by floods or storms, and with the formation of vulnerable and marginalized groups that lack the financial resources for adaptation, and may be forced to settle in climate-exposed areas (Adger et al. 2004). Thus, finding good-quality socioeconomic data at local scale and harmonizing them with climatic variables is a significant aspect of vulnerability research.

Hence, the purpose of this paper is to assess vulnerability to climate change at a local level in Ecuador. We rely on the statistical behavior of climatic and socioeconomic variables where the weighting and aggregation mechanism of the composite vulnerability indicator is similar to Iyengar and Sudarshan (1982) and Deepa et al. (2013). Rather than assuming the variables contribute equally to the composite indicator, our assessment approach defines the weights or contributions of each variables with respect to the aggregated variability of all variables across cantons (i.e. the political-administrative division of Ecuador, see the Additional file 1). Thus, through normalization, this approach mitigates the dominance of variables with large variance. Furthermore, it allows aggregation of variables to a measure of the overall variability that represents the multi-dimensionality of vulnerability (Leichenko and O’Brien 2002). We use a large number of economic, social, and environmental variables to construct the composite vulnerability indicator, and a contribution of this paper is the use of empirical orthogonal functions (EOF) and reanalysis datasets to incorporate the long-run and spatial patterns of the climatic variables.

We assume that exposure to climate change affects the sensitivity of cantons where communities respond given their adaptive capacity. Thus, we separate the composite indicator into three components: (1) exposure: the condition of disadvantage due to the position or location of a subject, object or system at risk; (2) sensitivity: the degree of internal ability of a subject, object or system to meet a threat and receive a possible impact due to the occurrence of an adverse event; and (3) adaptive capacity: the ability of a system, community or society exposed to hazards to cope, absorb, and recover from the effects of an adverse event effectively and in a timely manner, considering the preservation and restoration of its essential basic structures and functions (Antwi-Agyei et al. 2012; Houghton 1996; Ionescu et al. 2008; Luers et al. 2003; Martin et al. 2012; O’Brien et al. 2004).

The paper is organized as follows: “Methods” section describes both the modelling framework to construct the composite vulnerability indicator, and the data. “Results and discussion” section presents the results. “Conclusions” section concludes.

## Methods

We choose Ecuador as a case study because socioeconomic data are readily and publicly available. Also the cantons in this country show a large degree of heterogeneity in terms of development and climatic conditions. We map vulnerability indicators at the canton level and show how different factors that shape vulnerability vary within Ecuador. The dataset includes 221 cantons of the continental territory of Ecuador, and other settlements such as Manga del Cura, El Piedrero and Las Golondrinas, which, at the time the data were collected, did not belong to any province.

### Construction of the composite vulnerability indicator

Let $X_{id}$ denote the $i{\text{th}}$ vulnerability indicator in the $d{\text{th}}$ canton $({\text{i.e.}}\, i = 1,2, \ldots ,m;\;d = 1,2, \ldots n)$. We normalize each indicator such that $y_{id} = \frac{{X_{id} - Min_{d} X_{id} }}{{Max_{d} X_{id} - Min_{d} X_{id} }}$ if the indicator is assumed to be positively associated to vulnerability, or $y_{id} = \frac{{Max_{d} X_{id} - X_{id} }}{{Max_{d} X_{id} - Min_{d} X_{id} }}$ otherwise. From the matrix of normalized values, $Y_{{\left[ {n\;\times\;m} \right]}}$, the composite vulnerability indicator for the $d{\text{th}}$ canton is constructed as follows:

1 $$y_{d} = w_{1} y_{d1} + w_{2} y_{d2} + \cdots + w_{m} y_{dm}$$

$w_{i}$ is the weight of each indicator, that is, its contribution to the formation of the composite vulnerability indicator, where $0 < w_{i} < 1$ and $w_{1} + w_{2} + \cdots + w_{m} = 1$. To estimate the weights, we calculate the variance of each normalized indicator across cantons as $\sigma_{i}^{2} = \frac{{\mathop \sum \nolimits_{d = 1}^{n} \left( {y_{di} - \bar{y}_{i} } \right)^{2} }}{n - 1}$ where $\bar{y}_{i}$ is the average value. We define the weights relative to the aggregate behavior of the indicators variability across cantons, that is, weights vary inversely with the variance across the cantons as follows:

2 $$w_{i} = \frac{{\sqrt {\sigma_{i}^{2} } }}{{\mathop \sum \nolimits_{i = 1}^{m} \sqrt {\sigma_{i}^{2} } }}$$

This weighting mechanism ensures that large variation in any of the indicators will not dominate the contribution of the rest, and rules out the possibility that, for policy purposes, improvements in an indicator can perfectly compensate detriments in any other.

For the purposes of generality and meaningful ranking of the composite indicators we assume $y_{d}$ follows a Beta distribution in the range (0, 1) as $y_{d}$ is positive valued and potentially skewed (Iyengar 1982). The probability density of the Beta distribution is as follows:

3 $$f(z) = \frac{{z^{a - 1} (1 - z)^{b - 1} {\text{d}}x}}{B(a,b)},\quad 0 < z < 1\;{\text{and}}\; a,b > 0$$

where $$B(a,b) = \mathop \smallint \nolimits_{0}^{1} x^{a - 1} (1 - x)^{b - 1} {\text{d}}x.$$

The parameters $(a,b)$ can be estimated by solving the simultaneous equations:

4 $$(1 - y)a - yb = 0$$

5 $$(y - m)a - mb = m - y$$

where, $y$ is the overall mean of the composite indicators and $m$ is defined as:

6 $$m = s_{y}^{2} + y^{2}$$

where $s_{y}^{2}$ is the variance of the composite indicators.

In particular, if $y_{d}$ better fits a normal distribution, the parameters $a$ and $b$ will be equal (Vidwans 1983) so that a beta will approximate the normal distribution introducing non-negligible errors in the results (Pratt 1968). The opposite reasoning, however, may not apply, as assuming a normal distribution for variables with significant asymmetry would introduce errors in the statistical operations, in addition to specification problems.

Let $(0,z_{1} ), (z_{1} ,z_{2} ), (z_{2} ,z_{3} ), (z_{3} ,z_{4} ), (z_{4} ,z_{5} )$ be the linear intervals such that each one has the same probability weight of 20 %. These fractal groups classify cantons by vulnerability categories as follows:

Less vulnerable if $0 < y_{i} < z_{1}$.

Moderately vulnerable if $z_{1} < y_{i} < z_{2}$.

Vulnerable if $$z_{2} < y_{i} < z_{3}$$.

Highly vulnerable if $z_{3} < y_{i} < z_{4}$.

Very high vulnerability if $z_{4} < y_{i} < 1$.

For vulnerability assessments based on indicators, principal components analysis, encompassing all social and environmental variables, is an alternative to construct the composite vulnerability indicator. It has been widely used and is based on sound statistical theory (Gbetibouo et al. 2010; Leichenko and O’Brien 2002). The aim of PCA is to summarize information through the reduction of dimensions in such a way that the first principal component accounts for as much of the variability in the data as possible, and each succeeding component in turn has the highest variance possible under the constraint that it is orthogonal to the preceding components. PCA are guaranteed to be independent if the data set is jointly normally distributed (Jolliffe 2005). Though we do not intend our assessment approach to compete with PCA, we argue that PCA may exclude variables or indicators with relatively lower contributions to the formation of the components so that a comprehensive ordering of the indicators, as the one we intend in this paper, may not be achieved. The normality assumption in PCA should be managed with care for the same reasons we assume the beta distribution. Thus, PCA would not fit the purpose of this paper. Furthermore, we want to preserve the direct interpretation of results that may not exist when using the components derived from PCA. We therefore restrict the use of PCA for the construction of the EOF and the institutional indicators.

### Vulnerability indicators

From the mid-1990s the approach to vulnerability changed from an exclusive focus on meteorological and biophysical factors to a comprehensive approach that included social, economic, and political dimensions (Blaikie et al. 2004; Bohle et al. 1994; Cutter 1996; Kelly and Adger 2000). We separated the variables into the exposure, sensitivity and adaptive capacity indicators of each canton. For appropriate selection of the variables we held extensive discussions and a workshop with experts both in Ecuador and at the headquarters of the Inter-American Development Bank, Washington DC. Thus, we selected 42 variables and did not attempt to reduce dimensions (e.g. via principal components analysis) as our interest is to identify the effects of each variable on the formation of the composite indicator.

#### Climatic variables

Precipitation, temperature, relative humidity, and wind velocity are part of the essential climate variables (ECV) identified by the Global Climate Observing System (GCOS) as relevant for understanding the climatic system (Mason et al. 2010). Climatic data in Ecuador are spatially heterogeneous as a result of geophysical factors; however, their availability is limited. The coarse resolution of global circulation models (GCM) does not allow reliable geographic interpolations or aggregations. Thus, to incorporate the spatial and temporal pattern of the climatic indicators we use reanalysis datasets that are the outcome of numerical models and observational data assimilation with a finer resolution than the GCM.

The climatic variables come from the climate forecast system reanalysis (CFSR) developed by the National Center for Environmental Prediction (NCEP). This reanalysis model is the first that includes both oceanic and atmospheric features, and has demonstrated that it adequately reproduces precipitation and surface temperature, and calibrates for the natural and inter-seasonal variability (Saha et al. 2010; Tett et al. 1997). The ECVs are on a monthly basis for a range of 40 years (1971–2011) and are extracted in a rectangular grid (83 W–74 W, 2 N–6 S). The NCEP reanalysis dataset coincides with the climate observation satellite area, which implies that reliable observations are added in the assimilation process (Kanamitsu et al. 2002).

Four climatic indices were constructed to describe the trends of the climatic variables. The dataset was divided into two regions: western Ecuador (coastal zone) and eastern Ecuador (the Inter-Andean and Amazon regions). Thus, we implemented the EOF for the monthly variation of each climatic variable at canton level, except for precipitation, where we used the yearly averages because there were only 4 or 5 months with positive values of precipitation, whereas for the rest of the year the monthly values were equal to zero. This variation does not significantly affect the results. For computational purposes we use the EOF as the dimensions of reanalysis datasets are large since they combine time series with spatial maps. The EOF characterizes the temporal and spatial patterns of the climatic variables in the areas of interest, and have been used extensively in meteorological and climatological studies (Dai et al. 1997; Kutzbach 1967; Messié and Chavez 2011; Tatli and Türkeş 2011; von Storch and Navarra 1999). The EOF is equivalent to the principal components used in multivariate statistics and is close relative to the bases used in factor analysis. Furthermore, the EOF basis set defined directly from data can be used to represent a climatological field very economically (Kutzbach 1967), both as a basis for a set of predictors (Barnett and Hasselmann 1979) or as a means of physically interpreting the data (Wallace and Gutzler 1981). That is, if one thinks of the snapshots of geophysical data maps as realizations of random fields generated by a stochastic process, it is possible to construct second moment statistics linking one point and another in the map. The resulting covariance matrix is real and symmetric and therefore possesses a set of orthogonal eigenvectors with positive eigenvalues. The map associated with each eigenvector represents a pattern that is statistically independent of the others and spatially orthogonal to them. The eigenvalue indicates the amount of variance accounted for by the pattern. The eigenvector patterns contain information about the multidimensional probability distribution that constitutes the climate and are, therefore, of theoretical interest (North 1984). Thus, the EOF is designed to derive the dominant variability patterns from sets of fields of any type of synthetic indicators or indexes, and summarize the variability observed in a group of variables (Bultó et al. 2006; Thomson and Emery 2014).

#### Sensitivity and adaptive capacity indicators

Table 1 shows the socioeconomic indicators related to sensitivity and adaptive capacity. The indicators related to human capital, family structure, physical infrastructure, economic capability, socially vulnerable groups, demographics, and employment come from the National System of Indicators (SNI in Spanish) (http://www.sni.gob.ec). The SNI contains data from several national-level census and surveys administered in Ecuador. Housing tenure and housing characteristics information comes from the 2010 National Population and Household Census. Institutional capacity indicators are constructed based on the 2010 Census for the Management, Expenditures and Investment on Environmental Protection at Municipalities and Provincial Councils. Agricultural information such as the proportion of agricultural land with respect to total area, the proportion of agricultural land with irrigation, as well as tenure of agricultural land comes from the 2010 National Agricultural Census. Information about medical doctors and staff comes from the 2009 Health Resources and Activities Census.

**Table 1:** Vulnerability indicators

Focus	Indicators	Source
Sensitivity		
Demographics	Illiteracy rate (+)	NSI
	Population density (+)	NSI
	Unemployment rate (+)	NSI
Socially vulnerable groups	Average number of children per household (+)	NSI
	Proportion of crowded households (+)	NSI
	Proportion of population 0–5 years (+)	NSI
	Proportion of population 65 years or older (+)	NSI
	Proportion of population with permanent disability (+)	NSI
Land	Proportion agricultural land/total land extension (+)	NAC
	Proportion irrigated land/total agricultural land (−)	NSI
Adaptive capacity		
Physical infrastructure	Proportion of households receiving water through piped system (−)	NSI
	Proportion of households with access to computer (−)	NSI
	Proportion of households with electricity service (−)	NSI
	Proportion of households with garbage collection service (−)	NSI
	Proportion of households with land phone service (−)	NSI
	Proportion of households with proper sanitary facilities (−)	NSI
	Proportion of households with sewage treatment service (−)	NSI
	Proportion of houses with exclusive room for kitchen (−)	NSI
	Proportion of houses with exclusive sanitary facilities (−)	NSI
	Proportion of population with internet access (−)	NSI
	Proportion of population with mobile phone access (−)	NSI
	Minimum distance to large town (−)^a^	NSI
Economic capability	Average business revenues (−)	EC
	Average energy consumption (Kwh/annum) (−)	EC
	Average time in business (−)	EC
	Proportion of population working in own business (−) (amb)	NSI
	Proportion of households on agriculture owning land (+)	NSI
	Proportion of population working agriculture, hunting or fisheries (+)	NSI
	Tax revenues per capita (−)	NSI
Human capital	Hospital beds per capita (−)	HRAC
	Population per medical doctor (+)	HRAC
	Average number of years of scholarity for head of household (−)	NSI
	Proportion of households living on owned house (−)	NSI
	Proportion of households where head is female (+)	NSI
	Proportion of population under social security coverage (−)	NSI
	Proportion of population with private health insurance (−)	NSI
	Proportion of population affected by disasters (+)	DIMS
Institutional capacity	Funds for environmental protection per capita (−)	EMS
	Institutional capacity index (−)	EMS

From a conceptual standpoint (+) represents a positive relationship between the indicator and vulnerability, and (−) represents a negative relationship

*NSI* National System of Information 2010, *NAC* National Agricultural Census 2010, *EMS* Environmental Management Survey 2010, *HRAC* Health Resources and Activities Census 2009, *DIMS* Disaster Information Management System (Desinventar)

^a^Computed by the authors

To construct the institutional capacity indicator we apply polychoric principal component analysis to a set of variables related to whether the canton has developed information, programs, strategies or policies on: (1) environmental sensitivity assessments; (2) environmental development; (3) pollution management; and (4) climate change. Though these programs and policies are not uniform across cantons, their availability and extent are an input for the management of climate-related hazards and other environmental risks. Thus, the institutional indicator and its weight signal that in Ecuador the capacities and the relative institutional development of the cantons play a significant role in terms of resource management to cope with changing climatic conditions. We also include the per capita funds municipalities allocate or receive for investments on environmental protection.

As a proxy for the economic size of each canton we use indicators related to the average business revenues, average annual energy consumption of households, proportion of businesses with a national or foreign client, tax revenue per capita, and proportion of the population who work in their own business.

## Results and discussion

**Fig. 1:**
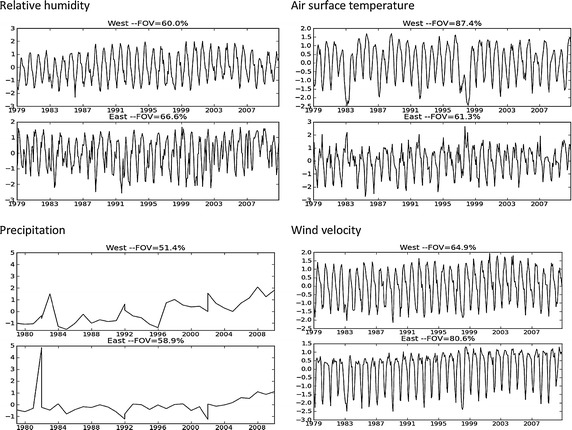
EOF/PC analysis of climatic indicators. Fraction of variance explained (FOV) for each climate indicator is greater than 51 %

Figure 1 shows the results of the EOF analysis. Each panel shows the first principal component (PC) and the share of variance explained. Temperature, wind velocity, and relative humidity show inter-annual variation, which may be interpreted as seasonal variation. Thus, correlating the temporal variation with the corresponding PC, at each location, quantifies the link between these climatic variables and the seasonal variations. Also, it is noticeable that these variables in western Ecuador show a better representation of the seasonal variation because the boundaries of this area include the significant climatic influences from the Pacific Ocean. The PCs of temperature, wind velocity, and relative humidity in eastern Ecuador represent the seasonal variation but with small perturbations as in this region there are significant orographic and vegetation differences that affect the behavior of these variables. Additionally, the PCs do not show a strong representation of extreme events such as El Niño or La Niña.

The EOF analysis of the monthly variation of precipitation resulted in a PC that did not show positive correlations. The monthly variation of precipitation is highly dispersed. For example, Northern Ecuador shows different values from the South because of the greater influence of the Intertropical Convergence Zone. This adds to the existing geophysical differences between the Eastern and Western zones. Hence, for EOF analysis of precipitation we use the yearly variation to obtain the PC at the geographic location of each of the cantons. As it may be observed in the first PC, both in Eastern and Western Ecuador, there is a strong signal at the occurrence period of events such as El Niño and La Niña in 1981 and 1984. The PCs explain 51.4 and 58.9 % of the yearly variation and result in positive correlations with the geographic locations of the cities.

### Vulnerability assessment

**Fig. 2:**
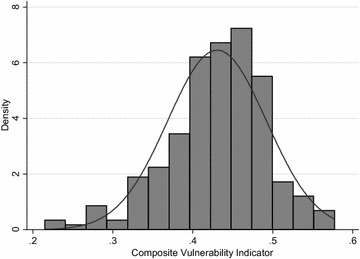
Plot of the composite vulnerability indicator

For the beta distribution, the parameter $a$ is equal to 26, and $b$ to 34. The cut-off points are *z*_*1*_ = 0.378, *z*_*2*_ = 0.415, *z*_*3*_ = 0.447 and *z*_*4*_ = 0.485. Figure 2 shows the plot of the composite vulnerability indicator. Although the asymmetry does not seem pronounced, the Skewness/Kurtosis tests reject the null hypothesis of normality (*p* value equal to 0.0005).

Table 2 shows the calculated weights of each indicator, that is, their contribution to the formation of the composite vulnerability indicators. The largest weight corresponds to the institutional capacity. For the relative ordering we observe that the climatic indicators are among the ten with the largest contributions. Temperature has the second largest contribution, then wind velocity has the third, relative humidity the sixth; and, precipitation the tenth. Other indicators with large contributions are the proportion of households with sewage treatment service; the proportion of households with garbage collection service; the proportion of population working in agriculture, hunting or fisheries; the proportion of households owning agricultural land; and, the proportion of households receiving water through a pipe system.

**Table 2:** Calculated weights of the vulnerability indicators

Institutional capacity	0.0484	Proportion of houses with exclusive sanitary facilities	0.0239
Temperature	0.0476	Proportion of population 65 years or older	0.0236
Wind velocity	0.0466	Proportion of crowded households	0.0230
Proportion of households with sewage treatment service	0.0321	Average number of children per household	0.0224
Proportion of households with garbage collection service	0.0319	Proportion of households with land phone service	0.0218
Relative humidity	0.0317	Illiteracy rate	0.0206
Proportion of population working agriculture, hunting or fisheries	0.0305	Proportion of population less than 5 year old	0.0203
Proportion of households on agriculture owning land	0.0297	Average time in business	0.0202
Proportion of households receiving water through piped system	0.0292	Proportion of population with mobile access	0.0202
Precipitation	0.0282	Average business revenues	0.0191
Proportion of population not under social security coverage	0.0279	Proportion of population with any permanent disability	0.0181
Proportion of households living on owned house	0.0272	Proportion of population with private health insurance	0.0168
Proportion of households below poverty line	0.0271	Proportion of households with electricity service	0.0168
Proportion irrigated land/total agricultural land	0.0258	Proportion of population affected by disasters	0.0149
Proportion of population with internet access	0.0258	Minimum distance to large town	0.0145
Proportion of households with access to computer	0.0256	Tax revenues per capita	0.0125
Net rate high school attendance	0.0255	Funds for environmental protection per capita	0.0118
Proportion of households with proper sanitary facilities	0.0253	Population per non-medical doctor	0.0106
Employment rate	0.0252	Population density	0.0102
Proportion of households where head is female	0.0240	Number of patients per hospital bed	0.0099
Proportion of population working in own business	0.0240	Average energy consumption (Kwh/annum)	0.0095

In turn, the indicators with the lowest contribution are funds for environmental protection per capita, population per non-medical doctor, population density, number of patients per hospital bed, and average energy consumption. Intermediate values appear for some of the sensitivity indicators such as the proportion of population with no social security coverage (11th), the proportion of households living below the poverty line (13th), the proportion of irrigated agricultural land with respect to total agricultural land (14th), the proportion of households with proper sanitary facilities (18th), and the proportion of households where the head is a female (20th).

**Fig. 3:**
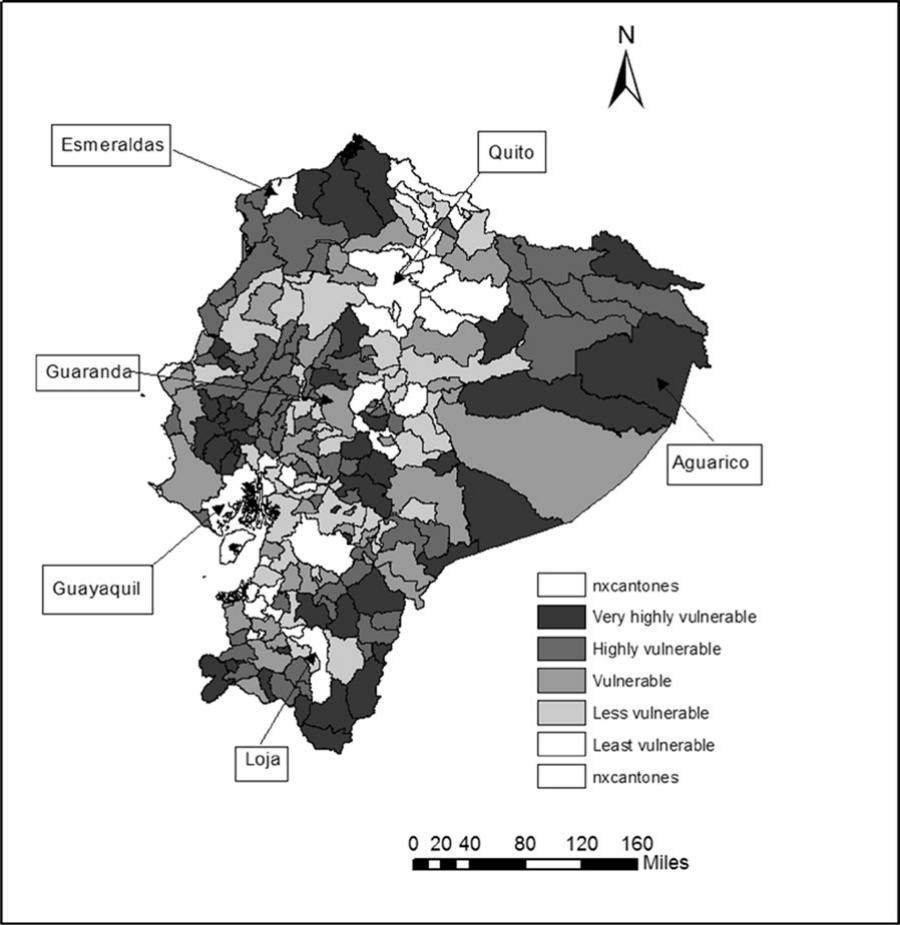
Vulnerability to climate change by Canton

Figure 3 shows the vulnerability categories. The cantons with the highest vulnerabilities are located to the northwest of Ecuador, in the province of Esmeraldas, to the Midwest, in the provinces of Manabi and Los Rios, and to the South, in the provinces of Loja and Morona Santiago. In addition, most of the cantons in the Amazonian region are either in the highly or very highly vulnerable categories. The least and less vulnerable cantons are located to the central region of the country, mainly in the provinces of Pichincha; others are in the South, in the province of El Oro. The least vulnerable cantons are Guayaquil, Ecuador’s largest city, and, Quito, Ecuador’s capital. These are followed by Cuenca, the third largest city in the country, and other mid-size cities such as Riobamba, Esmeraldas, Machala, Ambato, Loja, and Santo Domingo where populations range between 190,000 and 505,000 inhabitants. The correlation between the composite vulnerability indicator and population size is −0.5. This result points to the fact that larger cantons are not significantly less vulnerable.

Other smaller cantons, such as Mejia (pop. 81,335) and Marcelino Maridueña (pop. 12,033), are also in the less vulnerable categories. Arguably the proximity of Mejia to Quito and of Marcelino Maridueña to Guayaquil implies that the development facilities and infrastructure of large cities spill over to the adaptive capacity of surrounding smaller cantons. This situation may also explain the low vulnerability of other cantons such as Samborondon and Duran with respect to Guayaquil; Pasaje and Santa Rosa with respect to Machala; and Rumiñahui with respect to Quito. The situation does not, however, apply to cantons such as Salitre and Nobol, which despite their close proximity to Guayaquil (on average 20 miles), appear as highly vulnerable.

In the category of moderately vulnerable cantons we find that Quevedo, Latacunga, and Babahoyo (where population size is at least 153,000 inhabitants) are not different, in terms of vulnerability, from other much smaller cantons such as Calvas (pop. 28,185), Macara (pop. 19,018), San Pedro de Huaca (pop. 7624), and Chaguarpamba (7161). This indicates that there is no clear pattern between population size and vulnerability to climate change. In the category of vulnerable cantons we find Portoviejo (pop. 280,029), which is one of the 10 most populated cantons in the country, as well as smaller cantons like Guaranda (pop. 25,001), Salinas (pop. 28,650), and Quilanga (pop. 4337).

In the category of highly vulnerable cantons we have El Empalme (pop. 64,789), Santa Elena (pop. 30,920), Pallatanga (pop. 12,000) and Gualaquiza (pop. 7409). For the very highly vulnerable category we find mid-size cantons such as Lago Agrio (pop. 57,727), Pedro Carbo (pop. 31,337), Olmedo (pop. 4870), and Aguarico (pop. 1024), which is the canton with the highest vulnerability index.

### Decomposition of the vulnerability indicator

**Fig. 4:**
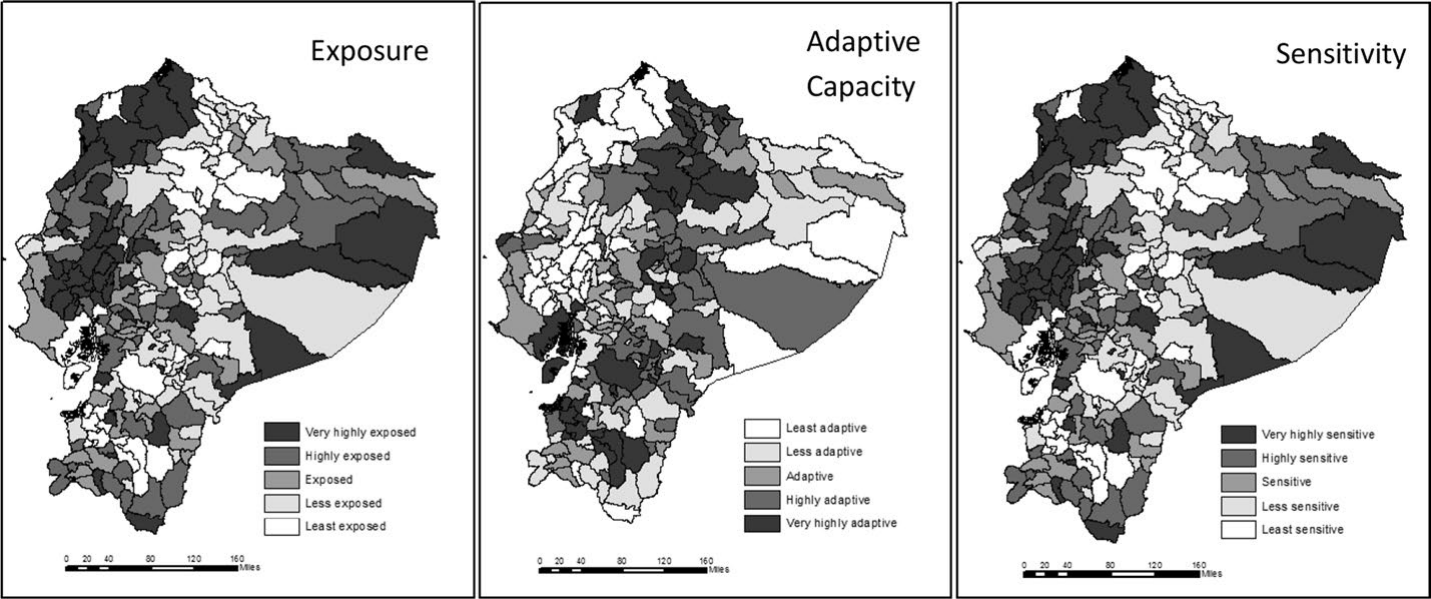
Components of vulnerability

Figure 4 shows the mapping of the components of vulnerability, that is, exposure, sensitivity, and adaptive capacity. Cantons with the lowest adaptive capacity are located in the Northwest of Ecuador, in the province of Esmeraldas; in the Midwest, in the provinces of Manabí and Guayas; and in the East, in the Amazonian provinces of Orellana and Pastaza. In turn, the cantons with very high adaptive capacity are Quito, Guayaquil, and Machala, where some of the surrounding cantons also belong to the very high adaptive capacity category.

The cantons with the highest exposure are also located in the Northwest of Ecuador, in the province of Esmeraldas, in the Midwest in the province of Manabi, and along the Amazonian region. The least exposed cantons are located mainly in the North and Central Regions of Ecuador, and Guayaquil, and some others to the South, in the provinces of Loja and Morona Santiago. The coastal zone is highly exposed, in particular, to the tropical monsoon climate (TMC), which extends to the Andean and Amazonian region. This climate pattern is regulated by atmospheric circulation and influences the seasonal changes of wind intensity and direction. Wind transports heat and moisture from the sea and produces variable effects in the Andean region. In addition, as a result of the elevation gradient of the Andean mountains the exposure of cantons in the Amazonian region is relatively low.

The cantons with the highest sensitivity are mainly located to the Northwest and Midwest of Ecuador, with some other cantons in the Amazonian region. The least sensitive cantons are Quito, Guayaquil, Machala, and their surrounding areas, and others in the central region of Ecuador.

An implication from Fig. 4 is that the ordering from the composite indicator is not preserved for all components. For instance, although Quito is ranked as one of the least vulnerable cantons, because of its high adaptive capacity and low sensitivity, it is identified as highly exposed to climatic features. The same applies to Guayaquil, which is very highly exposed to climate but is one of the least vulnerable cantons in the country. On the other hand, a canton such as Sozoranga, which is in the less sensitive and very highly adaptive capacity categories, is ranked as one of those less exposed to climatic features. Similarly, Rumiñahui, a canton with very low sensitivity and high adaptive capacity, is highly exposed to climate.

Approximately 20 % of the population (2.9 million individuals) in Ecuador lives in cantons with high and very high vulnerability mainly because of limitations on adaptive capacity. A similar proportion appears for the population living in highly and very highly sensitive cantons. However, 64 % of the population (9.3 million individuals) lives in cantons that are considered highly and very highly exposed to climatic phenomena.

### Descriptive results by vulnerability group

**Fig. 5:**
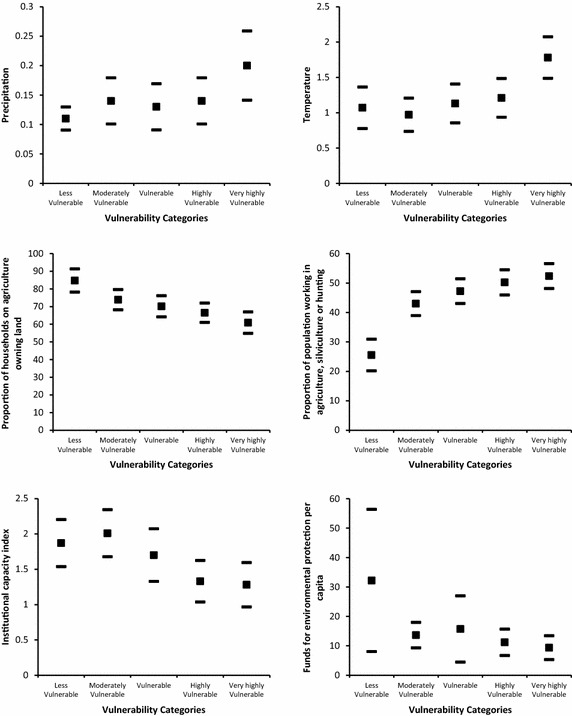
Descriptive results of selected indicators by vulnerability group. *Filled square* represents the average and *filled rectangle* represents upper and lower limits of the 95 % confidence interval

**Fig. 6:**
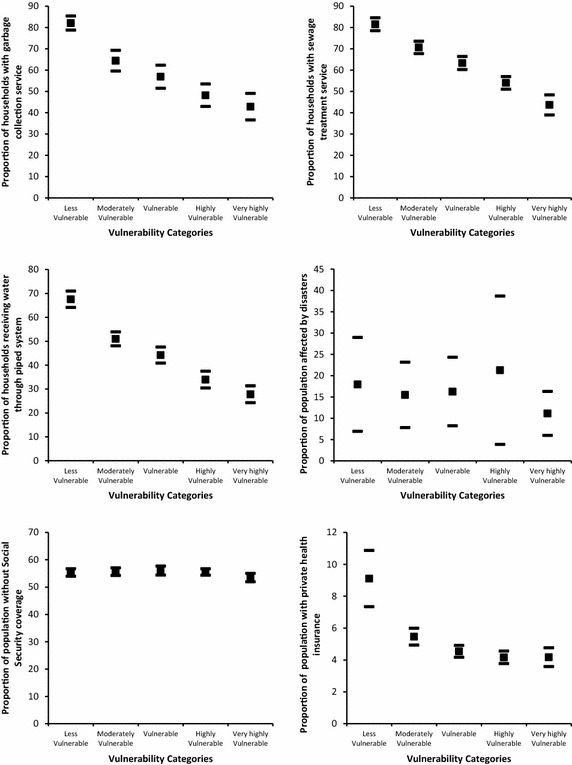
Descriptive results of selected indicators by vulnerability group. *Filled square* represents the average and *filled rectangle* represents upper and lower limits of the 95 % confidence interval

**Fig. 7:**
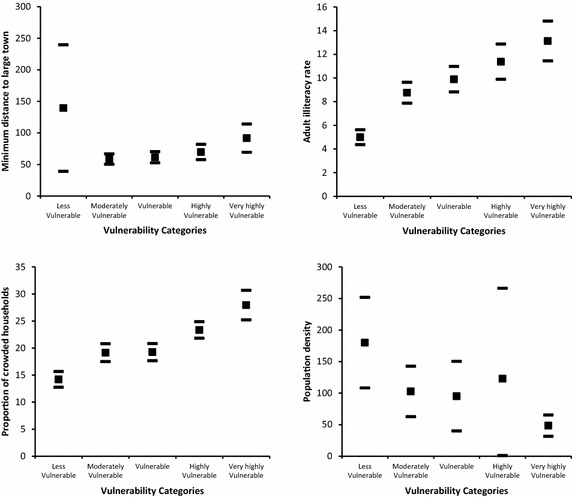
Descriptive results of selected indicators by vulnerability group. *Filled square* represents the average and *filled rectangle* represents upper and lower limits of the 95 % confidence interval

Figures 5, 6 and 7 shows the confidence intervals at 95 % level for selected indicators across vulnerability categories. For the climatic indicators we find that temperature and, to some extent, precipitation determine higher exposure in the very highly vulnerable category. Though in this category the confidence interval for precipitation overlaps with those in the moderately to highly vulnerable categories; temperature is significantly different compared with the rest of the vulnerability categories.

For the adaptive capacity indicators we find that agricultural land tenure (i.e. the proportion of households owning agricultural land with respect to total agricultural land) is not significantly different between the moderately vulnerable to the very highly vulnerable categories. In these groups on average 71 % of farmers own the land where they work. This figure increases to 85 % for the less vulnerable category. There is, however, a significant difference between the less and very highly vulnerable groups. In addition, for the proportion of the population working in agriculture, silviculture or hunting, 25.53 % work in cantons in the less vulnerable category. This figure increases to 52.35 % in the rest of the vulnerability categories.

The indicator for institutional capacity is highest for the moderately vulnerable category, and lowest for the highly and very highly vulnerable categories. Significant differences may appear in confidence intervals using levels slightly lower than 95 %. Public funds per capita for environmental protection are highest for the less vulnerable category (32.18 US dollars) but there is wide variation across cantons. For the rest of the categories funds per capita are on average 12.45 US dollars.

The proportion of households with access to garbage collection service is highest in the less vulnerable category (82.08 %). This is significantly different compared with the moderately vulnerable (64.43 %), the vulnerable (56.89 %), and the highly and very highly vulnerable (45.51 %) categories. Similarly, on average the proportion of households with sewage treatment service is 81.55 % in the less vulnerable category but is 43.67 % in the very highly vulnerable category. A similar result is observed for the proportion of households receiving water through a public piped system: the proportion is 67.54 % in the less vulnerable category, 33.95 % in the highly vulnerable category, and 27.8 % in the very highly vulnerable.

Regarding human capital as part of adaptive capacity, there is high variation in the proportion of population affected by natural disasters, where the average is 16.54 %. No significant differences are found across vulnerability categories. In addition, less than 10 % of the population across all vulnerability categories has private health insurance: 55 % of the population is not under social security coverage.

There is also a high variation on the minimum distance to a large town in the less vulnerable category and the confidence intervals overlap across all categories.

Regarding the sensitivity indicators, adult illiteracy rate is significantly higher in the very highly vulnerable category (13.12 %), and lower in the less vulnerable (4.99 %) and moderately vulnerable (8.75 %) categories. For the remaining categories, the illiteracy rate averages 10.01 %. For crowded households, this proportion is lowest for the less vulnerable category (14.19 %) and is significantly different for the rest of the vulnerability categories (highly vulnerable 23.33 % and very highly vulnerable, 27.93 %). Population density is significantly larger in the less vulnerable category (180 inhabitants per square km) compared with the very highly vulnerable category. However, there is a high variation where no pattern is identified and confidence intervals overlap across all categories.

## Conclusions

Earlier work has documented the extensive range of tools to assess climate change vulnerability at local levels. The use of any tool depends on the research questions to be answered. Vulnerability assessments, through the indicators approach, provide an overview of the socioeconomic, climatic, and geophysical determinants for a canton, or any other geographic unit, that are vulnerable to climate change. In this paper we use an approach that enables the relative ordering of the cantons in the continental territory of Ecuador. Our aim is to make full use of the climatic and non-climatic features, and construct additional indicators related to exposure, sensitivity, and adaptive capacity to climate change (Pachauri et al. 2014). Our approach allows us to capture the multi-dimensionality of vulnerability in a comprehensive framework (Leichenko and O’Brien 2002).

To construct the composite vulnerability indicator we use a large number of variables. Thus, in order to attain a meaningful and theoretically consistent vulnerability indicator, we normalize the variables and calculate the weights based on their relative variability. The weights represent their contribution to the formation of the composite indicator.

As it was not possible to apply standard modelling procedures to climatic data, because of the data’s unavailability or coarse resolution, we use a reanalysis strategy where the EOF incorporates spatial and temporal patterns. The composite indicator is bounded between 0 and 1 and takes the form of ratios, thus we assume it follows a beta distribution. It may be argued that this assumption does not apply to every dataset and context, but if a priori we assumed a normal distribution we would lose generality and incur potential specification errors if asymmetries arose. By assuming a beta distribution we leave open the possibility of approximating the beta to a normal distribution in case the parameters $a$ and $b$ are approximately equal (Iyengar and Sudarshan 1982). The errors from this approximation are negligible (Pratt 1968).

The institutional capacity of the cantons has the largest contribution to the formation of the composite indicator. Though we did not observe significant differences across the vulnerability categories, we argue that the availability of environmental- and climate-change-related policies improve the adaptive capacity. These policies serve to anticipate the impacts of climate change and form potential responses. However, this indicator does not capture the nature and extent of those policies. For this we use as a proxy the funds per capita allocated to environmental protection. Results showed that there is a wide variation in the less vulnerable category, thus we cannot reach a conclusion that funds are significantly larger for the cantons in this category. However, getting allocation of funds in general from the central government is often the outcome of an intense lobbying process that depends on the relative economic and political importance of each canton. As the less vulnerable category includes some of Ecuador’s largest cantons (e.g. Guayaquil and Quito) along with others that are much smaller (e.g. Mejía and Marcelino Maridueña), we argue that correcting by economy and population size, both institutional capabilities and greater funds for environmental development can determine whether a canton is less vulnerable to climate change.

Earlier work has shown that variables such as the proportion of households with sewage treatment service, with garbage collection service, and with access to water through piped system indicate both poverty levels and development (Baker 2004; Feitelson and Chenoweth 2002). Thus, we found that those cantons in the least vulnerable category are also those with higher coverage of these public services. In addition, the proportion of population working in agriculture, hunting or fisheries and the proportion of households on owning agricultural land are associated with local economic capabilities and opportunities. Results show that these indicators are among the ten largest contributions to the composite indicator. That is, vulnerability in Ecuador is also influenced by the concentration of economic activities related to agriculture, which limits other economic activities and the ability of people to shift in response to reduced agricultural income that may result from adverse climatic conditions. Some implications of these results arise: first, land tenure promotes economic development and reduces vulnerability as economic deprivation, in an adverse climatic event, is partially resolved by selling land assets. In addition, as agricultural work does not necessarily demand academic qualifications or schooling, this affects human capital accumulation and implies reduced adaptive capacity.

For cantons in the highly and very highly vulnerable categories, at least 50 % of the population works in agriculture, hunting or fisheries. With a weaker contribution to the composite indicator, the proportion of agricultural land that is irrigated has the fourteenth largest contribution. To this we add that 64 % of Ecuador’s population (9.3 million individuals) lives in cantons that are considered highly and very highly exposed. Thus, small holder and subsistence farmers will suffer impacts of climate change that will be locally specific and hard to predict. Hence, the variety of crop and livestock species produced by any one household, and their interactions in production and marketing, will increase the complexity both of the impacts and of subsequent adaptations (Morton 2007). In addition, approximately 20 % of the population (2.9 million individuals) live in cantons with high and very high vulnerability mainly because of the limitations of adaptive capacity.

Some of the largest cantons in Ecuador (Quito, Guayaquil, and Machala) show a very high adaptive capacity, given their development infrastructure, which may spill into surrounding cantons. However, it is not possible to conclude that there is a strong correlation between population size and adaptive capacity because mid-size cantons such as El Empalme and Santa Elena are categorized as highly vulnerable. Furthermore, we cannot generalize that proximity to the largest cantons will guarantee coverage of public services, for example, Salitre and Nobol, which, despite their close proximity to Guayaquil (on average 20 miles), appear as highly vulnerable.

For policy purposes, particular attention should be directed to the low coverage of health services, private health insurance, and social security. Though coverage may have increased since data collection, it may not be at a high enough level to guarantee adequate adaptive capacity in most of the cantons analysed.

Some limitations are worth mentioning. First, we do not include the Galapagos Islands, as their climatic features are different from Ecuadorian continental territory. Second, though we do not intend to compete with other vulnerability approaches and have not developed a formal comparison test with PCA, a research path would be to assess how different weighting of indicators influence interpretation and to identify the potential links to planning, prioritization, decision-making, and monitoring over time, given the dynamic nature of vulnerability (Bele et al. 2013). Third, we did not take a particular focus on agriculture or rural areas. Future economic or demographic impact analysis would certainly complement the extent of the implications of this study.

## Additional file

10.1186/s40064-015-1536-z Political-administrative division of Ecuador.

## References

[CR1] Adger WN, Brooks N, Bentham G, Agnew M (2004). New indicators of vulnerability and adaptive capacity.

[CR2] Antwi-Agyei P, Fraser EDG, Dougill AJ, Stringer LC, Simelton E (2012). Mapping the vulnerability of crop production to drought in Ghana using rainfall, yield and socioeconomic data. Appl Geogr.

[CR3] Baker J (2004) Analyzing urban poverty: a summary of methods and approaches, vol 3399. World Bank Publications

[CR4] Barnett TP, Hasselmann K (1979). Techniques of linear prediction, with application to oceanic and atmospheric fields in the tropical Pacific. Rev Geophys.

[CR5] Bele MY, Tiani AM, Somorin OA, Sonwa DJ (2013). Exploring vulnerability and adaptation to climate change of communities in the forest zone of Cameroon. Clim Change.

[CR6] Blaikie P, Cannon T, Davis I, Wisner B (2004) At risk: natural hazards, people’s vulnerability and disasters. Routledge, p 89

[CR7] Bohle HG, Downing TE, Watts MJ (1994). Climate change and social vulnerability. Glob Environ Change.

[CR8] Brenkert AL, Malone EL (2005). Modeling vulnerability and resilience to climate change: a case study of India and Indian states. Clim Change.

[CR9] Bultó PLO, Rodríguez AP, Valencia AR, Vega NL, Gonzalez MD, Carrera AP (2006). Assessment of human health vulnerability to climate variability and change in Cuba. Environ Health Perspect.

[CR10] Cutter SL (1996). Vulnerability to environmental hazards. Prog Human Geogr.

[CR11] Dai A, Fung IY, Del Genio AD (1997). Surface observed global land precipitation variations during 1900–88. J Clim.

[CR12] Deepa B, Hiremath DB, Shiyani RL (2013). Analysis of vulnerability indices in various agro-climatic zones of Gujarat. Anal.

[CR13] Eakin H (2005). Institutional change, climate risk, and rural vulnerability: cases from Central Mexico. World Develop.

[CR15] Feitelson E, Chenoweth J (2002). Water poverty: towards a meaningful indicator. Water Policy.

[CR16] Füssel H-M (2010). How inequitable is the global distribution of responsibility, capability, and vulnerability to climate change: a comprehensive indicator-based assessment. Glob Environ Change.

[CR17] Füssel H-M, Klein RJT (2002) Assessing vulnerability and adaptation to climate change: an evolution of conceptual thinking. In: UNDP Expert Group Meeting on 'Integrating disaster reduction and adaptation to climate change'. Havana, pp 17–19

[CR18] Füssel H-M, Klein RJT (2006). Climate change vulnerability assessments: an evolution of conceptual thinking. Clim Change.

[CR19] Gbetibouo GA, Ringler C, Hassan R (2010). Vulnerability of the South African farming sector to climate change and variability: an indicator approach. Nat Resour Forum.

[CR20] Houghton JT (1996) Climate change 1995: the science of climate change: contribution of working group I to the second assessment report of the intergovernmental panel on climate change, vol 2. Cambridge University Press, p 572

[CR21] Ionescu C, Klein RJT, Hinkel J, Kavi Kumar KS, Klein R (2008). Towards a formal framework of vulnerability to climate change. Environ Model Assess.

[CR22] Iyengar NS, Sudarshan P (1982). A method of classifying regions from multivariate data. Econ Political Wkly.

[CR23] Jolliffe I, Everitt BS, Howell DC (2005). Principal component analysis. Encyclopedia of statistics in behavioral science.

[CR24] Kanamitsu M, Ebisuzaki W, Woollen J, Yang S-K, Hnilo JJ, Fiorino M, Potter GL (2002). NCEP–DOE AMIP-II Reanalysis (R-2). Bull Am Meteorol Soc.

[CR25] Kelly PM, Adger WN (2000). Theory and practice in assessing vulnerability to climate change. Clim Change.

[CR26] Kutzbach JE (1967). Empirical eigenvectors of sea-level pressure, surface temperature and precipitation complexes over North America. J Appl Meteorol.

[CR27] Leichenko RM, O’Brien KL (2002). The dynamics of rural vulnerability to clobal change: the case of southern Africa. Mitig Adapt Strateg Glob Change.

[CR28] Luers AL, Lobell DB, Sklar LS, Addams CL, Matson PA (2003). A method for quantifying vulnerability, applied to the agricultural system of the Yaqui Valley, Mexico. Glob Environ Change.

[CR29] Martin R, Bachelet B, Hill DRC, Bellocchi G, Lardy R (2012) Ecosystem climate change vulnerability assessment framework. In: International Congress on Environmental Modelling and Software Managing Resources of a Limited Planet, pp 777–784

[CR30] Mason PJ, Zillman JW, Simmons A, Lindstrom EJ, Harrison DE, Dolman H, Rasmussen J (2010) Implementation plan for the global observing system for climate in support of the UNFCCC (2010 Update). Geneva, WMO, IOC, UNEP, ICSU, pp 180

[CR31] Mendelsohn R, Dinar A, Williams L (2006). The distributional impact of climate change on rich and poor countries. Environ Develop Econ.

[CR32] Messié M, Chavez F (2011). Global modes of sea surface temperature variability in relation to regional climate indices. J Clim.

[CR33] Morton JF (2007). The impact of climate change on smallholder and subsistence agriculture. Proc Natl Acad Sci USA.

[CR34] North GR (1984). Empirical orthogonal functions and normal modes. J Atmos Sci.

[CR35] O’Brien K, Leichenko R, Kelkar U, Venema H, Aandahl G, Tompkins H, West J (2004). Mapping vulnerability to multiple stressors: climate change and globalization in India. Glob Environ Change.

[CR36] Pachauri RK, Allen MR, Barros VR, Broome J, Cramer W, Christ R, Church JA et al (2014) Climate Change 2014 In: Synthesis Report. Contribution of Working Groups I, II and III to the Fifth Assessment Report of the Intergovernmental Panel on Climate Change, pp 151

[CR37] Pandey R, Jha S (2011). Climate vulnerability index—measure of climate change vulnerability to communities: a case of rural Lower Himalaya, India. Mitig Adapt Strateg Glob Change.

[CR38] Parry M, Rosenzweig C, Iglesias A, Livermore M, Fischer G (2004). Effects of climate change on global food production under SRES emissions and socio-economic scenarios. Glob Environ Change.

[CR39] Pratt J (1968). A normal approximation for binomial, F, Beta, and other common, related tail probabilities, II. J Am Stat Assoc.

[CR40] Saha S, Moorthi S, Pan H-L, Wu X, Wang J, Nadiga S, Goldberg M (2010). The NCEP climate forecast system reanalysis. Bull Am Meteorol Soc.

[CR41] Schilling J, Freier KP, Hertig E, Scheffran J (2012). Climate change, vulnerability and adaptation in North Africa with focus on Morocco. Agric Ecosyst Environ.

[CR42] Sutanta H, Rajabifard A, Bishop ID (2013). Disaster risk reduction using acceptable risk measures for spatial planning. J Environ Plan Manage.

[CR43] Tatli H, Türkeş M (2011). Empirical orthogonal function analysis of the palmer drought indices. Agric Forest Meteorol.

[CR44] Tett S, Johns T, Mitchell J (1997). Global and regional variability in a coupled AOGCM. Clim Dyn.

[CR14] Thomson RE, Emery WJ (2014) Data analysis methods in physical oceanography, 3rd edn. Newnes, Elsevier, P 87

[CR45] Tol RSJ (2002). Estimates of the damage costs of climate change, part II. Dynamic estimates. Environ Resour Econ.

[CR46] Vidwans SM (1983). A method of classifying regions from multivariate data. Econ Political Wkly.

[CR47] Von Storch H, Navarra A (eds) (1999) Analysis of climate variability: applications of statistical techniques. Springer Science and Business Media, pp 100

[CR48] Wallace JM, Gutzler DS (1981). Teleconnections in the geopotential height field during the Northern Hemisphere Winter. Month Weather Rev.

[CR49] Watson RT, Zinyowera MC, Moss RH (1998) The regional impacts of climate change: an assessment of vulnerability. Cambridge University Press, Cambridge, UK, p 517

