# Supplementary material for: Assessing local vulnerability to climate change in Ecuador
Source: Springerplus. 2015 Nov 26;4:738. doi: 10.1186/s40064-015-1536-z (PMC4661167; doi:10.1186/s40064-015-1536-z)
Supplement: Supplementary file 1 — 10.1186/s40064-015-1536-z Political-administrative division of Ecuador. [file 40064_2015_1536_MOESM1_ESM.docx]

Figure S1: Provinces of Ecuador


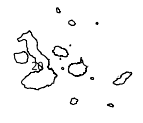

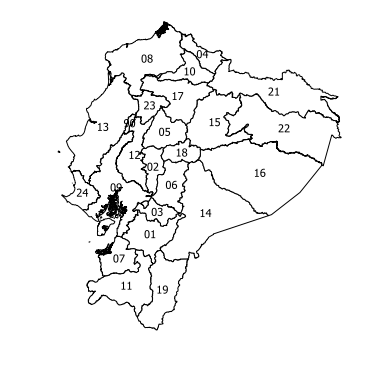


| Code | Provinces |
| --- | --- |
| 1 | Azuay |
| 2 | Bolivar |
| 3 | Cañar |
| 4 | Carchi |
| 5 | Cotopaxi |
| 6 | Chimborazo |
| 7 | El Oro |
| 8 | Esmeraldas |
| 9 | Guayas |
| 10 | Imbabura |
| 11 | Loja |
| 12 | Los Rios |
| 13 | Manabi |
| 14 | Morona Santiago |
| 15 | Napo |
| 16 | Pastaza |
| 17 | Pichincha |
| 18 | Tungurahua |
| 19 | Zamora Chinchipe |
| 20 | Galapagos |
| 21 | Sucumbios |
| 22 | Orellana |
| 23 | Santo Domingo de los Tsachilas |
| 24 | Santa Elena |

Figure S2: Cantons in Western Ecuador


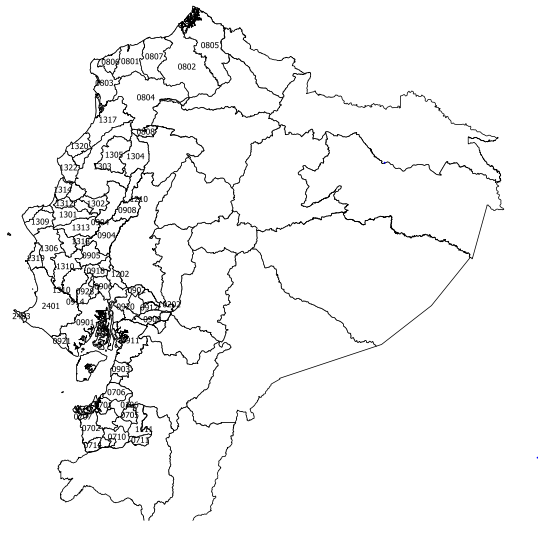


| **Canton Code** | **Canton** | **Province** |
| --- | --- | --- |
| 701 | MACHALA | EL ORO |
| 709 | PASAJE | EL ORO |
| 712 | SANTA ROSA | EL ORO |
| 706 | EL GUABO | EL ORO |
| 713 | ZARUMA | EL ORO |
| 703 | ATAHUALPA | EL ORO |
| 705 | CHILLA | EL ORO |
| 710 | PIÑAS | EL ORO |
| 704 | BALSAS | EL ORO |
| 711 | PORTOVELO | EL ORO |
| 707 | HUAQUILLAS | EL ORO |
| 714 | LAS LAJAS | EL ORO |
| 702 | ARENILLAS | EL ORO |
| 708 | MARCABELI | EL ORO |
| 802 | ELOY ALFARO | ESMERALDAS |
| 805 | SAN LORENZO | ESMERALDAS |
| 803 | MUISNE | ESMERALDAS |
| 806 | ATACAMES | ESMERALDAS |
| 801 | ESMERALDAS | ESMERALDAS |
| 808 | LA CONCORDIA | ESMERALDAS |
| 807 | RIOVERDE | ESMERALDAS |
| 804 | QUININDE | ESMERALDAS |
| 902 | ALFREDO BAQUERIZO MORENO | GUAYAS |
| 903 | BALAO | GUAYAS |
| 921 | PLAYAS | GUAYAS |
| 911 | NARANJAL | GUAYAS |
| 907 | DURAN | GUAYAS |
| 901 | GUAYAQUIL | GUAYAS |
| 920 | SAN JACINTO DE YAGUACHI | GUAYAS |
| 909 | EL TRIUNFO | GUAYAS |
| 910 | MILAGRO | GUAYAS |
| 912 | NARANJITO | GUAYAS |
| 922 | SIMON BOLIVAR | GUAYAS |
| 923 | CRNEL. MARCELINO MARIDUEÑA | GUAYAS |
| 927 | GNRAL. ANTONIO ELIZALDE | GUAYAS |
| 908 | EMPALME | GUAYAS |
| 914 | PEDRO CARBO | GUAYAS |
| 928 | ISIDRO AYORA | GUAYAS |
| 924 | LOMAS DE SARGENTILLO | GUAYAS |
| 925 | NOBOL | GUAYAS |
| 918 | SANTA LUCIA | GUAYAS |
| 906 | DAULE | GUAYAS |
| 919 | SALITRE | GUAYAS |
| 916 | SAMBORONDON | GUAYAS |
| 905 | COLIMES | GUAYAS |
| 913 | PALESTINA | GUAYAS |
| 904 | BALZAR | GUAYAS |
| 908 | EMPALME | GUAYAS |
| 904 | BALZAR | GUAYAS |
| 921 | PLAYAS | GUAYAS |
| 914 | PEDRO CARBO | GUAYAS |
| 928 | ISIDRO AYORA | GUAYAS |
| 1311 | PICHINCHA | MANABI |
| 1313 | SANTA ANA | MANABI |
| 1318 | OLMEDO | MANABI |
| 1317 | PEDERNALES | MANABI |
| 1311 | PICHINCHA | MANABI |
| 1315 | TOSAGUA | MANABI |
| 1302 | BOLIVAR | MANABI |
| 1322 | SAN VICENTE | MANABI |
| 1320 | JAMA | MANABI |
| 1304 | EL CARMEN | MANABI |
| 1303 | CHONE | MANABI |
| 1305 | FLAVIO ALFARO | MANABI |
| 1306 | JIPIJAPA | MANABI |
| 1310 | PAJAN | MANABI |
| 1313 | SANTA ANA | MANABI |
| 1316 | 24 DE MAYO | MANABI |
| 1318 | OLMEDO | MANABI |
| 1319 | PUERTO LOPEZ | MANABI |
| 1308 | MANTA | MANABI |
| 1309 | MONTECRISTI | MANABI |
| 1312 | ROCAFUERTE | MANABI |
| 1307 | JUNIN | MANABI |
| 1301 | PORTOVIEJO | MANABI |
| 1321 | JARAMIJO | MANABI |
| 1314 | SUCRE | MANABI |
| 1310 | PAJAN | MANABI |
| 2401 | SANTA ELENA | SANTA ELENA |
| 2402 | LA LIBERTAD | SANTA ELENA |
| 2403 | SALINAS | SANTA ELENA |
| 9004 | EL PIEDRERO | ZONA NO DELIMITADA |
| 9003 | MANGA DEL CURA | ZONA NO DELIMITADA |

Figure S3: Cantons in Central Ecuador


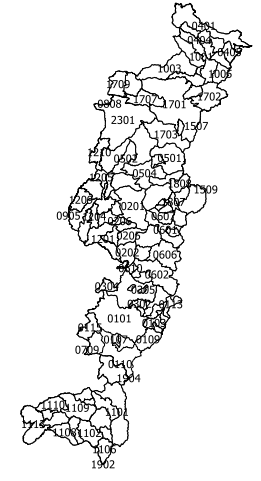


| **Canton Code** | **Canton** | **Province** |
| --- | --- | --- |
| 101 | CUENCA | AZUAY |
| 102 | GIRON | AZUAY |
| 103 | GUALACEO | AZUAY |
| 104 | NABON | AZUAY |
| 105 | PAUTE | AZUAY |
| 106 | PUCARA | AZUAY |
| 107 | SAN FERNANDO | AZUAY |
| 108 | SANTA ISABEL | AZUAY |
| 109 | SIGSIG | AZUAY |
| 110 | OÑA | AZUAY |
| 111 | CHORDELEG | AZUAY |
| 112 | EL PAN | AZUAY |
| 113 | SEVILLA DE ORO | AZUAY |
| 114 | GUACHAPALA | AZUAY |
| 115 | CAMILO PONCE ENRIQUEZ | AZUAY |
| 201 | GUARANDA | BOLIVAR |
| 202 | CHILLANES | BOLIVAR |
| 203 | CHIMBO | BOLIVAR |
| 204 | ECHEANDIA | BOLIVAR |
| 205 | SAN MIGUEL | BOLIVAR |
| 206 | CALUMA | BOLIVAR |
| 207 | LAS NAVES | BOLIVAR |
| 301 | AZOGUES | CAÑAR |
| 302 | BIBLIAN | CAÑAR |
| 303 | CAÑAR | CAÑAR |
| 304 | LA TRONCAL | CAÑAR |
| 305 | EL TAMBO | CAÑAR |
| 306 | DELEG | CAÑAR |
| 307 | SUSCAL | CAÑAR |
| 401 | TULCAN | CARCHI |
| 402 | BOLIVAR | CARCHI |
| 403 | ESPEJO | CARCHI |
| 404 | MIRA | CARCHI |
| 405 | MONTUFAR | CARCHI |
| 406 | SAN PEDRO DE HUACA | CARCHI |
| 501 | LATACUNGA | COTOPAXI |
| 502 | LA MANA | COTOPAXI |
| 503 | PANGUA | COTOPAXI |
| 504 | PUJILI | COTOPAXI |
| 505 | SALCEDO | COTOPAXI |
| 506 | SAQUISILI | COTOPAXI |
| 507 | SIGCHOS | COTOPAXI |
| 601 | RIOBAMBA | CHIMBORAZO |
| 602 | ALAUSI | CHIMBORAZO |
| 603 | COLTA | CHIMBORAZO |
| 604 | CHAMBO | CHIMBORAZO |
| 605 | CHUNCHI | CHIMBORAZO |
| 606 | GUAMOTE | CHIMBORAZO |
| 607 | GUANO | CHIMBORAZO |
| 608 | PALLATANGA | CHIMBORAZO |
| 609 | PENIPE | CHIMBORAZO |
| 610 | CUMANDA | CHIMBORAZO |
| 704 | BALSAS | EL ORO |
| 708 | MARCABELI | EL ORO |
| 709 | PASAJE | EL ORO |
| 713 | ZARUMA | EL ORO |
| 714 | LAS LAJAS | EL ORO |
| 808 | LA CONCORDIA | ESMERALDAS |
| 901 | GUAYAQUIL | GUAYAS |
| 905 | COLIMES | GUAYAS |
| 916 | SAMBORONDON | GUAYAS |
| 923 | CRNEL. MARCELINO MARIDUEÑA | GUAYAS |
| 927 | GNRAL. ANTONIO ELIZALDE | GUAYAS |
| 1001 | IBARRA | IMBABURA |
| 1002 | ANTONIO ANTE | IMBABURA |
| 1003 | COTACACHI | IMBABURA |
| 1004 | OTAVALO | IMBABURA |
| 1005 | PIMAMPIRO | IMBABURA |
| 1006 | SAN MIGUEL DE URCUQUI | IMBABURA |
| 1101 | LOJA | LOJA |
| 1102 | CALVAS | LOJA |
| 1103 | CATAMAYO | LOJA |
| 1104 | CELICA | LOJA |
| 1105 | CHAGUARPAMBA | LOJA |
| 1106 | ESPINDOLA | LOJA |
| 1107 | GONZANAMA | LOJA |
| 1108 | MACARA | LOJA |
| 1109 | PALTAS | LOJA |
| 1110 | PUYANGO | LOJA |
| 1111 | SARAGURO | LOJA |
| 1112 | SOZORANGA | LOJA |
| 1113 | ZAPOTILLO | LOJA |
| 1114 | PINDAL | LOJA |
| 1115 | QUILANGA | LOJA |
| 1116 | OLMEDO | LOJA |
| 1201 | BABAHOYO | LOS RIOS |
| 1202 | BABA | LOS RIOS |
| 1203 | MONTALVO | LOS RIOS |
| 1204 | PUEBLOVIEJO | LOS RIOS |
| 1205 | QUEVEDO | LOS RIOS |
| 1206 | URDANETA | LOS RIOS |
| 1207 | VENTANAS | LOS RIOS |
| 1208 | VINCES | LOS RIOS |
| 1209 | PALENQUE | LOS RIOS |
| 1210 | BUENA FE | LOS RIOS |
| 1211 | VALENCIA | LOS RIOS |
| 1212 | MOCACHE | LOS RIOS |
| 1213 | QUINSALOMA | LOS RIOS |
| 1403 | LIMON INDANZA | MORONA SANTIAGO |
| 1405 | SANTIAGO | MORONA SANTIAGO |
| 1406 | SUCUA | MORONA SANTIAGO |
| 1501 | TENA | NAPO |
| 1504 | EL CHACO | NAPO |
| 1507 | QUIJOS | NAPO |
| 1509 | CARLOS JULIO AROSEMENA TOLA | NAPO |
| 1701 | QUITO | PICHINCHA |
| 1702 | CAYAMBE | PICHINCHA |
| 1703 | MEJIA | PICHINCHA |
| 1704 | PEDRO MONCAYO | PICHINCHA |
| 1705 | RUMIÐAHUI | PICHINCHA |
| 1707 | SAN MIGUEL DE LOS BANCOS | PICHINCHA |
| 1708 | PEDRO VICENTE MALDONADO | PICHINCHA |
| 1709 | PUERTO QUITO | PICHINCHA |
| 1801 | AMBATO | TUNGURAHUA |
| 1802 | BAÑOS DE AGUA SANTA | TUNGURAHUA |
| 1803 | CEVALLOS | TUNGURAHUA |
| 1804 | MOCHA | TUNGURAHUA |
| 1805 | PATATE | TUNGURAHUA |
| 1806 | QUERO | TUNGURAHUA |
| 1807 | SAN PEDRO DE PELILEO | TUNGURAHUA |
| 1808 | SANTIAGO DE PILLARO | TUNGURAHUA |
| 1809 | TISALEO | TUNGURAHUA |
| 1902 | CHINCHIPE | ZAMORA CHINCHIPE |
| 1904 | YACUAMBI | ZAMORA CHINCHIPE |
| 2105 | SUCUMBIOS | SUCUMBIOS |
| 2301 | SANTO DOMINGO | SANTO DOMINGO DE LOS TSACHILAS |

Figure S4: Cantons in Eastern Ecuador


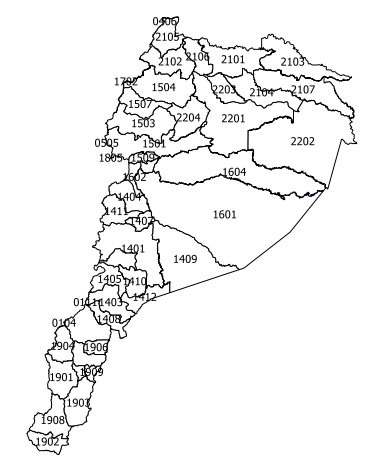


| **Canton Code** | **Canton** | **Province** |
| --- | --- | --- |
| 103 | GUALACEO | AZUAY |
| 104 | NABON | AZUAY |
| 110 | OÑA | AZUAY |
| 111 | CHORDELEG | AZUAY |
| 112 | EL PAN | AZUAY |
| 401 | TULCAN | CARCHI |
| 402 | BOLIVAR | CARCHI |
| 405 | MONTUFAR | CARCHI |
| 406 | SAN PEDRO DE HUACA | CARCHI |
| 501 | LATACUNGA | COTOPAXI |
| 505 | SALCEDO | COTOPAXI |
| 601 | RIOBAMBA | CHIMBORAZO |
| 604 | CHAMBO | CHIMBORAZO |
| 1111 | SARAGURO | LOJA |
| 1401 | MORONA | MORONA SANTIAGO |
| 1402 | GUALAQUIZA | MORONA SANTIAGO |
| 1403 | LIMON INDANZA | MORONA SANTIAGO |
| 1404 | PALORA | MORONA SANTIAGO |
| 1405 | SANTIAGO | MORONA SANTIAGO |
| 1406 | SUCUA | MORONA SANTIAGO |
| 1407 | HUAMBOYA | MORONA SANTIAGO |
| 1408 | SAN JUAN BOSCO | MORONA SANTIAGO |
| 1409 | TAISHA | MORONA SANTIAGO |
| 1410 | LOGROÐO | MORONA SANTIAGO |
| 1411 | PABLO SEXTO | MORONA SANTIAGO |
| 1412 | TIWINTZA | MORONA SANTIAGO |
| 1501 | TENA | NAPO |
| 1503 | ARCHIDONA | NAPO |
| 1504 | EL CHACO | NAPO |
| 1507 | QUIJOS | NAPO |
| 1509 | CARLOS JULIO AROSEMENA TOLA | NAPO |
| 1601 | PASTAZA | PASTAZA |
| 1602 | MERA | PASTAZA |
| 1603 | SANTA CLARA | PASTAZA |
| 1604 | ARAJUNO | PASTAZA |
| 1701 | QUITO | PICHINCHA |
| 1702 | CAYAMBE | PICHINCHA |
| 1805 | PATATE | TUNGURAHUA |
| 1901 | ZAMORA | ZAMORA CHINCHIPE |
| 1902 | CHINCHIPE | ZAMORA CHINCHIPE |
| 1903 | NANGARITZA | ZAMORA CHINCHIPE |
| 1904 | YACUAMBI | ZAMORA CHINCHIPE |
| 1905 | YANTZAZA | ZAMORA CHINCHIPE |
| 1906 | EL PANGUI | ZAMORA CHINCHIPE |
| 1907 | CENTINELA DEL CONDOR | ZAMORA CHINCHIPE |
| 1908 | PALANDA | ZAMORA CHINCHIPE |
| 1909 | PAQUISHA | ZAMORA CHINCHIPE |
| 2101 | LAGO AGRIO | SUCUMBIOS |
| 2102 | GONZALO PIZARRO | SUCUMBIOS |
| 2103 | PUTUMAYO | SUCUMBIOS |
| 2104 | SHUSHUFINDI | SUCUMBIOS |
| 2105 | SUCUMBIOS | SUCUMBIOS |
| 2106 | CASCALES | SUCUMBIOS |
| 2107 | CUYABENO | SUCUMBIOS |
| 2201 | ORELLANA | ORELLANA |
| 2202 | AGUARICO | ORELLANA |
| 2203 | LA JOYA DE LOS SACHAS | ORELLANA |
| 2204 | LORETO | ORELLANA |
